# Scaling up tobacco cessation within TB programmes: findings from a multi-country, mixed-methods implementation study

**DOI:** 10.1186/s12961-022-00842-1

**Published:** 2022-04-18

**Authors:** Helen Elsey, Zunayed Al Azdi, Shophika Regmi, Sushil Baral, Razia Fatima, Fariza Fieroze, Rumana Huque, Jiban Karki, Dost Mohammad Khan, Amina Khan, Zohaib Khan, Jinshuo Li, Maryam Noor, Abriti Arjyal, Prabin Shrestha, Safat Ullah, Kamran Siddiqi

**Affiliations:** 1 Department of Health Sciences, University of York, Heslington, Y010 5DD UK; 2 ARK Foundation, Suite C-3, C-4, House # 06, Road # 109, Gulshan-2, Bangladesh; 3 HERDi, Prasuti Griha Marg, Kathmandu, 44600 Nepal; 4 Common Management Unit (TB, HIV/AIDS & Malaria), Islamabad, Pakistan; 5 TB Control Programme, Peshawar, Khyber Pakhtunkhwa Province Pakistan; 6 The Initiative, Orange Grove Farm, Main Korung Road, Malpur, Bani Gala, Islamabad, Pakistan; 7 Khyber Medical University, F1 Phase-6 Rd, Phase 5 Hayatabad, Peshawar, 25100 Khyber Pakhtunkhwa Pakistan

**Keywords:** Scale-up, Tuberculosis, Tobacco cessation, Implementation research, ExpandNet steps for scale-up

## Abstract

**Background:**

Brief behavioural support can effectively help tuberculosis (TB) patients quit smoking and improve their outcomes. In collaboration with TB programmes in Bangladesh, Nepal and Pakistan, we evaluated the implementation and scale-up of cessation support using four strategies: (1) brief tobacco cessation intervention, (2) integration of tobacco cessation within routine training, (3) inclusion of tobacco indicators in routine records and (4) embedding research within TB programmes.

**Methods:**

We used mixed methods of observation, interviews, questionnaires and routine data. We aimed to understand the extent and facilitators of vertical scale-up (institutionalization) within 59 health facility learning sites in Pakistan, 18 in Nepal and 15 in Bangladesh, and horizontal scale-up (increased coverage beyond learning sites). We observed training and surveyed all 169 TB health workers who were trained, in order to measure changes in their confidence in delivering cessation support. Routine TB data from the learning sites were analysed to assess intervention delivery and use of TB forms revised to report smoking status and cessation support provided. A purposive sample of TB health workers, managers and policy-makers were interviewed (Bangladesh *n* = 12; Nepal *n* = 13; Pakistan *n* = 19). Costs of scale-up were estimated using activity-based cost analysis.

**Results:**

Routine data indicated that health workers in learning sites asked all TB patients about tobacco use and offered them cessation support. Qualitative data showed use of intervention materials, often with adaptation and partial implementation in busy clinics. Short (1–2 hours) training integrated within existing programmes increased mean confidence in delivering cessation support by 17% (95% CI: 14–20%). A focus on health system changes (reporting, training, supervision) facilitated vertical scale-up. Dissemination of materials beyond learning sites and changes to national reporting forms and training indicated a degree of horizontal scale-up. Embedding research within TB health systems was crucial for horizontal scale-up and required the dynamic use of tactics including alliance-building, engagement in the wider policy process, use of insider researchers and a deep understanding of health system actors and processes.

**Conclusions:**

System-level changes within TB programmes may facilitate routine delivery of cessation support to TB patients. These strategies are inexpensive, and with concerted efforts from TB programmes and donors, tobacco cessation can be institutionalized at scale.

## Background

A detrimental relationship between tuberculosis (TB) and tobacco use is well established. Smoking is responsible for 16% of the global TB disease burden by adversely affecting TB outcomes [1–5]. Brief behaviour support for tobacco cessation delivered by health professionals as part of routine TB care has been found to be effective in helping patients to quit [6, 7] and improving TB outcomes [8]. Despite evidence of its effectiveness, this simple and affordable intervention has yet to be implemented and scaled-up within national TB programmes (NTPs) in high-TB burden countries such as Bangladesh, Nepal and Pakistan [9]. This is consistent with the global picture where only 32% of the general population are able to access cessation services [10]. This is despite the high proportion of current tobacco smokers in all three countries: 41.4% of males and 1.4% of females in Bangladesh, 33.7% of males and 8.8% of females in Nepal and 31% of males and 2.8% of females in Pakistan [11]. In addition to high tobacco prevalence, all three countries suffer a high burden of TB incidence: 221 per 100,000 in Bangladesh, 2018 [12], 245 per 100,000 in Nepal, 2020 [13] and 265 per 100,000 in Pakistan, 2018 [12].

In Bangladesh, Nepal and Pakistan, despite increasing recognition by policy-makers of the need for tobacco cessation, the lack of health system components to support cessation such as recording and reporting, low health worker skill level and limited time per patient restrict its delivery [14–16]. Within pragmatic trials, where these health system barriers can be addressed in the short term, high quit rates have been achieved. The ASSIST randomized controlled trial (RCT) in Pakistan found 41.0% (95% CI, 37.1–45.0%) of those with suspected TB were able to quit at 6 months, verified biochemically, following brief behavioural support [6].

Overcoming health system barriers to allow scale-up of tobacco cessation within routine TB programmes has the potential to benefit patients' recovery from TB and their own future health outcomes across multiple noncommunicable diseases, as well as reducing the risks of second-hand smoking and risk of TB transmission among those around them [17]. Given the health systems challenges identified, this paper presents an implementation research study to understand what steps can be taken to implement “systems-orientated scale-up” [18] of brief behaviour support within routine TB care in Bangladesh, Nepal and Pakistan.

There has been an increased focus on developing and using frameworks to support systematic scale-up of health interventions [19–22]. ExpandNet’s *Nine steps for developing a scaling-up strategy* provides a useful framework to both plan and analyse the process [20] (see Table 5 for details of the nine steps) and defines scale-up as “deliberate efforts to increase the impact of successfully tested health innovations so as to benefit more people and to foster policy and programme development on a lasting basis.” (ExpandNet 2010 p. 9). ExpandNet distinguishes between vertical scale-up, that is, institutionalization of the intervention, and horizontal scale-up, that is, increased coverage. We drew on evidence of health systems challenges to integration of cessation support, careful discussion with TB programme managers and decision-makers following the ExpandNet process and existing evidence of what works when scaling-up public health interventions [19, 21, 23] to develop strategies for scale-up and to analyse the process of scale-up and any resulting vertical and horizontal scale-up of tobacco cessation within Bangladesh, Nepal and Pakistan. We worked with NTPs to identify learning sites in each country and to prioritize health system changes needed to support and facilitate the scale-up of tobacco cessation within routine services. Four strategies were identified, implemented and evaluated in each country and are described below.

### Strategies for scale-up

*Strategy 1: A simple and adaptable intervention* Step 1 of the ExpandNet framework focuses on intervention adaptation to ensure it is amenable to scale-up (see Table 5). Through a process of qualitative interviews, focus groups and workshops with TB managers, patients and health workers as reported elsewhere [9], content of the intervention used in the ASSIST [Action to Stop Smoking in Suspected Tuberculosis] trial [6] was condensed to a less than 10-minute consultation whilst retaining core behaviour change techniques (BCTs) [24] identified as effective in the trial [6]. TB health workers’ preference for a single resource that covered TB treatment messages, information on staying healthy (diet, rest, exercise) and tobacco cessation was adopted [25]. The components of the final simplified intervention are detailed in Table 1 below.

**Table 1:** TB & Tobacco intervention components and messages

Materials	Content	Messages and BCT taxonomy code [24]
*Flipbook:* for use during consultations with all TB patients following diagnosis and at any point during the 6 months of TB treatment to reinforce messages and support quit attempts	Eight pages with photo pages facing patients and text facing health professionals. One version with photos targeting male patients and one version with photos targeting female patients: the first 5 pages include key messages on TB management, and the final 3 pages have messages regarding tobacco (cigarettes and smokeless)	*TB messages:* (i) It is very likely that your TB will be cured if you take your medicines as instructed (ii) Keep taking medicines regularly (iii) Come for scheduled appointments, your health worker is here to support you (iv) Understanding how TB spreads (v) Importance of social support (vi) Adopt a healthy diet and lifestyle, including quitting tobacco *Cessation messages:* (i) Abrupt cessation: set a quit date and then, “not a puff” (ii) How to deal with side effects (iii) Identifying triggers and alternative strategies (iv) Consequences of tobacco use (cigarettes and smokeless) on TB, long-term health and finances (v) Dangers of second-hand smoke *Behaviour change techniques:* (i) Goal-setting (1.1) (ii) Reducing negative emotions (1.2) (iii) Action-planning (1.4) (iv) Prompting social support (3.1, 3.2, 3.3) (v) Instructions on performing behaviours (4.1) (vi) Information on health and emotional consequences (5.1, 5.6) (vii) Habit formation (8.3) (viii) Comparative imagining of future outcomes (9.3) (ix) Reducing negative emotions (11.2) (x) Reducing exposure to cues for behaviour (12.3) (xi) Building rapport, being an active listener
*Leaflet:* for TB patients, their carers and family members	Using photos and illustrations and simple text highlighting the consequences of tobacco use (cigarettes and smokeless), link with TB, benefits of quitting and how to deal with side effects of quitting	
*Two posters*: for use within TB clinic waiting areas	One presenting health benefits of quitting (general, not TB-specific) and one advertising the cessation service	
*A health worker guide*: to be used in conjunction with the flipbook, leaflet and posters	Providing the evidence behind the key messages, tips for adaptation and strategies for building rapport and good communication with both male and female patients	
Further adaptations made based on TB & Tobacco trial process evaluation		
*Desk reminder:* one page, laminated to be stuck on or beside the TB health worker’s desk	Reiterating key messages and including details of the TB & Tobacco website where all materials are available for further reference	
*Training of trainers (ToT)*: Half-day programme	Introducing the intervention, key messages and underlying evidence. How to deliver the messages and support TB health workers to deliver Includes a country-specific 10-minute video modelling how to ask about tobacco use, advise and support patients to quit	
*Training session*: 2-hour training session for TB health workers delivered by trainers following the ToT above	Introducing the intervention and key cessation messages, and how to complete the tobacco columns in the recording forms. Including the 10-minute video above	By delivering these key messages using simple BCTs, TB health workers help their patients to quit as part of routine care
*Awareness-raising video:* for TB programme managers and policy-makers	Three-minute video explaining the link between TB and tobacco and the need to include tobacco cessation support for TB patients	Inclusion of tobacco cessation within the TB programme is feasible and can improve TB outcomes

All training and intervention materials are freely available in Urdu, Bengali, Nepalese and English from the TB & Tobacco website: https://tbandtobacco.org/

*Strategy 2: Integration of cessation within routine training* Intense training over several days has been a feature of previous cessation interventions [6, 7]. Such resource-intensive exercises conducted outside regular, routine programme training can be unfeasible when attempting to scale up. However, strengthening the capacity of the implementing organization is a key ExpandNet step (see Table 5), and low health worker confidence in delivering cessation support has been identified previously [14, 15]. Our second strategy was to train a core of NTP trainers using the intervention materials with supporting videos (see Table 1) to roll out cessation training to TB health workers as part of routine programme training.

*Strategy 3: Including tobacco use in recording, monitoring and supervision* To highlight the priority given to tobacco cessation and track implementation, TB managers recommended adding tobacco use to TB reporting forms and NTP indicators. We held discussions with the central TB programmes to revise national TB reporting forms and redesigned existing TB reporting forms for use in the learning sites to include space to record for each patient (i) the smoking status at start of treatment, (ii) whether cessation advice had been given and (iii) smoking status at end of treatment.

*Strategy 4: Embedding research within TB programmes* TB & Tobacco Consortium researchers in all three countries drew on approaches to embed research for health policy and system change as recommended by Olivier et al. (2017) [26] and specified within the WHO 2012 strategy on health policy and systems research [27], by (1) using “insider researchers”, (2) working with NTPs in the design of the study and identification of learning sites, (3) immersion of researchers within the health system through participation in existing fora, planning events and regular meetings at the national and subnational levels and (4) identifying windows of opportunity for meaningful engagement.

Having agreed on these strategies with the TB programmes, this study aimed to understand the extent and processes of vertical scale-up (institutionalization) and horizontal scale-up (increased coverage).

*Context* Within many high-burden countries, including in Bangladesh, Nepal and Pakistan, TB diagnosis and treatment has been integrated within all levels of care in the health system using the Directly Observed Treatment, Short-course (DOTS) process [28] of 6 months of treatment for patients with drug-sensitive TB (i.e. TB that can be treated with first-line medication rather than drug-resistant TB which requires multiple medicines over a longer period of time). In Pakistan, TB care is integrated within the primary healthcare system which consists of a mix of public and private providers. Within the federal context, the programme is implemented by the district health authorities with the support of provincial TB control programmes and national leadership from the Common Unit for TB, AIDS and Malaria (CU). In Bangladesh, the National TB Control Programme, under the Directorate General of Health Services, manages the programme centrally, delivering TB care through a mix of public, private and nongovernmental organization (NGO) providers. Following constitutional reform in 2015, Nepal introduced a three-tier federal system, devolving management of TB services to local (municipality) level, with support from provincial health directorates and the National TB Control Centre at the federal level. A mix of public, private and NGO providers deliver TB care integrated within the primary healthcare system.

## Methods

We used an implementation science approach, where data collection occurs within routine practice in close collaboration with policy-makers and NTBs, often drawing on routinely collected data and allowing flexibility to enable exploration of real-life implementation [29]. Our study was based on a convergent mixed-methods design, combining quantitative and qualitative findings at the analysis stage [30] to understand the extent and nature of scale-up against the four strategies to understand vertical (institutionalization) and horizontal (increased coverage) scale-up of the tobacco cessation intervention.

*Learning sites* The selection of the learning sites was determined by NTPs based on local capabilities and interest to implement. In Pakistan, the national programme invited all provinces to participate. Khyber Pakhtunkhwa Province (KPP) volunteered to implement the intervention in 59 facilities out of the 121 in the province, selected to include urban, rural, public and private settings. In Nepal, one NGO-run community hospital, two NGO-run TB referral centres and 15 public primary care facilities within the Kathmandu and Lalitpur districts were selected. In Bangladesh, 15 facilities—12 Upazilla health complexes and three district hospitals from three districts—were selected to cover both rural and urban settings (see Table 2).

**Table 2:** Characteristics of learning sites

Total facilities	Hospitals	Primary healthcare clinics
Bangladesh (15)	3 Public	12 Public
Nepal (18)	3 NGO	15 Public
Pakistan (59)		
District 1: Peshawar (28)	9 Private/NGO 9 Public	4 Public 6 Private/NGO
District 2: Kohat (10)	2 Public	6 Public 2 Private/NGO
District 3 Abbottabad (9)	5 Public	2 Public 2 Private
District 4: Madan (12)	6 Public 1 Private/NGO	4 Public 1 Private

### Quantitative methods

As an indication of the likelihood of vertical scale-up within the TB programme, we assessed the impact of training on health worker confidence in delivering the intervention, drawing on the COM-B model [24]. Participants were asked to complete a pretested questionnaire [9] to assess capacity, opportunity and motivation for provision of cessation support before and after training. The pre- and post-training questionnaires were collected on paper and recorded in Excel. We also recorded the number of trainers trained and any subsequent training provided to TB health workers in the learning sites or beyond.

To assess the implementation of the intervention within the facilities (vertical scale-up), we collected routine data from the revised NTP reporting forms for all drug-sensitive TB patients 15 years and older in the three countries. We collected data for a period of 6 months from January to June 2019. However, only 3 months of data (April–June 2019) were collected in Nepal and two districts in Bangladesh due to delays in conducting the training to fit with existing NTP training schedules. Each site was visited at least once to observe implementation, assess completeness of recording and to collect routine TB data including the new tobacco use and support data. In Pakistan, due to the higher number of sites, observations were made in a purposive sample of 10 facilities (five public and five private of various sizes), and the routine TB and new tobacco data were collected from the district offices. The data collected included (1) the total number of drug-sensitive TB patients 15 years and older, (2) whether their smoking status was recorded and (3) whether they had received cessation advice. To assess correct usage of the new reporting columns, we estimated the proportion of appropriately completed data entry cells for the columns on tobacco use and tobacco support for all adult TB patients. To aid comparability, the total number of patients and the monthly mean number of patients are presented (see Table 6).

*Costs* We used an activity-based cost-analysis approach [31] to estimate the cost of implementation of the intervention per drug-sensitive TB patient (15 years and over) in learning sites. We collected data on (1) personnel salaries, fees and time taken in programme-related activities, including intervention delivery and training; (2) printing and disseminating programme/intervention materials; and (3) number of intervention sessions delivered. Costs did not include venue utilities (water, electricity, etc.), administrative activities (preparation meetings, organization contacts, etc.), logistics (stationery, refreshments, etc.) or salary on-costs (contribution to pension, health insurance, etc.) if applicable.

*Qualitative methods* TB & Tobacco Consortium researchers, native to each country and with experience in qualitative methods and the health system contexts, observed training delivery and the implementation of the intervention in each facility and wrote up observation reports. The observations were conducted at least once in all the sampled facilities in Nepal and Bangladesh in a purposive sample of 10 facilities in Pakistan (five public and five private of various sizes). These observations focused on patients’ interaction, use of the materials and completion of the reporting forms. Health workers and managers were interviewed to understand the facilitators and barriers to both vertical and horizontal scale-up. Training sessions were observed using a structured guide to record the length and content of the training, use of intervention materials and videos, use of interactive methods such as role-play, and quality of training provided. Given our aim to understand varied perspectives, we purposively sampled health workers, managers and policy-makers for semi-structured qualitative interviews from public, private and NGO facilities within the learning sites and TB programme managers at municipality, district, provincial and national level (Nepal *n* = 13; Pakistan *n* = 19; Bangladesh *n* = 12; see Table 3). All interviews were audio-recorded and translated into English. To determine the size of the interview sample, we drew on principles of “information power” [32], including ensuring that (1) the purposively selected interview participants brought in-depth knowledge relevant to the research aim and (2) the use of the Consolidated Framework for Implementation Research (CFIR) [33] to structure the interview guides and the analysis of qualitative interview and observation data. The five constructs and their subdomains within the CFIR enabled us to develop a comprehensive understanding of implementation, including how aspects of the cessation *intervention* itself, the *inner setting* of TB programmes, the *outer context*, *characteristics of individuals*, particularly TB staff at all levels, and the *process* used in each country. To better understand this last aspect, the research team used the nine steps of the ExpandNet framework [20] to reflect on the process we had taken and to identify any lessons learned (see Table 5).

**Table 3:** Characteristics of qualitative participants

ID	Sex	Type/level of organization	Designation
Nepal			
NP1	M	National TB programme	Technical officer
NP2	M	National TB programme	Technical officer
NP3	F	Municipality office	Technical officer
NP4	M	Public TB facility	Facility manager
NP5	F	District health office	TB health worker
NP6	F	NGO TB facility	Senior manager
NP7	F	Public TB facility	TB health worker
NP8	F	Public TB facility	TB health worker
NP9	F	Public TB facility	TB health worker
NP10	F	Public TB facility	TB health worker
NP11	F	Public TB facility	TB health worker
NP12	F	Public TB facility	TB health worker
NP13	F	NGO TB facility	TB health worker
Pakistan			
PK1	M	Public TB facility	TB facility manager
PK2	M	Private TB facility	TB health worker
PK3	M	Private TB facility	TB health worker
PK4	M	Public TB facility	TB health worker
PK5	M	Private TB facility	TB health worker
PK6	M	Private TB facility	TB health worker
PK7	F	Public TB facility	TB health worker
PK8	M	District government	Technical officer
PK9	M	Private TB facility	TB health worker
PK10	M	District government	Senior manager
PK11	M	Public TB facility	TB health worker
PK12	M	Public TB facility	TB health worker
PK13	M	Public TB facility	TB health worker
PK14	M	Private TB facility	TB health worker
PK15	M	Private TB facility	TB health worker
PK16	M	Private TB facility	TB health worker
PK17	M	Provincial government	Senior manager
PK18	M	Public TB facility	TB health worker
PK19	M	Private TB facility	TB health worker
Bangladesh			
BD1	M	Public TB facility	TB health worker
BD2	M	Public TB facility	TB health worker
BD3	F	Public TB facility	TB health worker
BD4	M	Public TB facility	TB health worker
BD5	M	Public TB facility	TB health worker
BD6	M	Public TB facility	TB health worker
BD7	M	Public TB facility	TB health worker
BD8	F	National TB programme	Senior manager
BD9	F	National TB programme	Senior manager
BD10	M	District TB programme	Senior manager
BD11	M	District TB programme	Technical officer
BD12	M	Public TB facility	Senior manager

*Analysis* The questionnaire data were analysed using descriptive statistics and the paired *t*-test in Stata version 16.1 to identify any significant changes in individual responses before and after the training. The routine facility data were analysed to identify proportions for each indicator. Given the different time periods of data collection, monthly mean patient numbers were calculated. The reported number of smokers identified were compared to the expected number of smokers given the age-adjusted prevalence of current tobacco smoking estimates provided by WHO based on 2017 data [11], and 95% confidence intervals around these ratios were calculated.

Costs were collected in the respective countries’ currencies—Bangladeshi taka (BDT), Nepalese rupee (NPR), Pakistani rupee (PKR)—and converted to US dollars (9 May 2019 price: USD 1.0 = BDT 84.5 = NPR 112.1 = PKR 142.0). As 3 months of data were collected from some learning sites, for comparison, the number of patients and smokers identified over 3 months was doubled to match observations over 6 months. The total costs in the learning sites were calculated and divided by the total TB patients reported in the observation period to derive a per-patient cost.

A framework approach [34] was used to structure the analysis against the CFIR constructs, with further codes added to align findings with our four strategies. Transcripts of the first two interviews conducted in each country were double-coded to enable reflection on the interview process and to refine the interview guide to further explore concepts of the CFIR. Consistency of use of the CFIR by the country research teams was enabled through a 3-day analysis workshop.

The methods used to understand progress made against each strategy are summarised in Table 4. The Standards for Reporting Implementation Studies (StaRI) guidelines have been followed in the reporting of this study [35].

**Table 4:** Data sources and analysis used to assess each of the four strategies

Strategy	Data source	Data analysed
Strategy 1: Simple and adaptable intervention	Routine facility data from 59 health facility learning sites in Pakistan, 18 in Nepal and 15 in Bangladesh	Proportion of drug-sensitive TB patients asked and advised about tobacco
	Interviews with TB health workers, managers, policy-makers (Bangladesh *n* = 12; Nepal *n* = 13; Pakistan *n* = 19)	Interview transcripts coded according to CFIR constructs and aligned to ExpandNet steps
	Researcher observations in 10 facilities in Pakistan, 18 in Nepal, 15 in Bangladesh	Facility observation reports to understand extent and nature of implementation
Strategy 2: Integration of cessation within routine training	Pre- and post-training questionnaires of TB health workers (169)	Significant changes in confidence to offer cessation support between pre- and post-training questionnaires
	Training observation reports	Number of trainers trained Any subsequent training provided to TB health workers in the learning sites or beyond
Strategy 3: Including tobacco use in recording, monitoring and supervision	Routine facility data from 59 health facility learning sites and observation of 10 in Pakistan. Routine data and observations from 18 facilities in Nepal and 15 in Bangladesh	Proportion of tobacco status, and advised columns completed appropriately
	Interviews with TB health workers, managers, policy-makers (Bangladesh *n* = 12; Nepal *n* = 13; Pakistan *n* = 19)	Interview transcripts coded according to CFIR constructs and aligned to ExpandNet steps
Strategy 4: Embedding research within the TB programmes	Cost data: salaries, fees, time taken in intervention activities, printing and disseminating materials; number of intervention sessions delivered	Activity-based cost analysis
	Interviews with TB health workers, managers and policy-makers (Bangladesh *n* = 12; Nepal *n* = 13; Pakistan *n* = 19)	Interview transcripts coded according to CFIR constructs and aligned to ExpandNet steps
	Reflections of the research team	Research team reflections aligned to ExpandNet steps

### Findings

Our findings are presented against each of the four systems-orientated scale-up strategies. The activities used to implement all four strategies are mapped against the ExpandNet nine steps in Table 5. The table also provides a summary of the lessons learned, drawing on the qualitative findings and reflections of the research team.

**Table 5:** Mapping TB & Tobacco Consortium actions to ExpandNet scale-up steps and framework

ExpandNet nine steps to scale-up	TB & Tobacco Consortium actions in relation to ExpandNet’s “CORRECT”^a^ intervention attributes to enable scale-up and principles of enhanced scalability, systems thinking, sustainability, equity	Lessons learned and reflections of TB & Tobacco Consortium actions
Step 1: Planning actions to increase the scalability of the innovation	Adaptation of cessation intervention found effect through research in South Asia (O, C) Engagement with national, provincial, regional TB programmes to adapt intervention and throughout the project (R) Reduced intervention content for shorter 10-minute cessation consultations (Co) Adding one-page desk guide for easy reference (E) Policy and guideline review (R)	RCT evidence from Pakistan and Bangladesh (7) of the effectiveness of the 10-minute cessation consultation enhanced the credibility of the intervention for policy-makers and practitioners, particularly as evidence showed the relative advantage over pharmaceutical interventions or no quit support (6, 48) Facilitating TB health workers to adapt and apply the intervention to their context helped with relevance, ownership and compatibility Policy review highlighted limited attention to tobacco cessation in existing policies and plans, indicating that although there is no comprehensive tobacco cessation programme in Bangladesh, Pakistan or Nepal, concern that felt need/relevance may not be great
Step 2: Building the capacity of the user organization for scale-up	Training trainers in the TB health system (Co) Working with NTPs to identify NTP staff to train as trainers (R) Designing short training sessions for use in routine TB programme (Co) with videos to maintain quality and consistency (C) Assessing capacity, opportunity and motivation of TB health professionals (T)	Developing and filming videos provided an opportunity for further engagement of NTP staff and TB health workers at all levels increased buy-in of TB programmes; e.g., Nepal’s NTP director introduced the training video Filming in TB clinics and modelling real-life consultations enhanced relevance Challenging to identify trainers likely to subsequently train others; e.g. in Nepal, eight trainers trained, but only two delivered training to TB health workers
Step 3: Assessing the environment and planning actions to increase the potential for scaling-up success	Identifying health system levers for vertical and horizontal scale-up (Co) Engagement with NTPs at national (Bangladesh), national and provincial (Pakistan) and municipalities (Nepal) to identify learning sites (R, O, T) Redesign of NTP recording and reporting forms (Co, T) Redesign of NTP supervision forms (Co) Supporting and assessing the delivery of training of health workers and dissemination of materials (Co, T)	Implementation research (IR) studies (14–16), workshops with TB managers, health workers, supervisors and research team’s in-depth knowledge of TB programmes in all three countries vital to identify health system levers Adapting existing reporting forms and guidelines and training programmes rather than developing parallel systems enhanced compatibility but was challenging to implement Despite increases in COM-B questionnaire scores following the short training, demand for longer training and regular refreshers highlighted that training is also seen as an incentive In general, NTP supervision in all three countries focused on checking reporting forms rather than support to provide quality care. Greater early engagement of first-line supervisors (e.g. programme officers in Bangladesh) in the future could help address this
Step 4: Increasing the capacity of the resource team to support scaling up	In-country research teams build relationships with NTPs (R, Co) Extended periods of research through multiple studies with national TB programmes (O, R, Co) Co-I and TB focal point as “insider-researchers” in Pakistan (C, R, Co)	Senior members of the research team have long-standing relationships with NTP at policy and programme levels and mutual respect developed through engagement in multiple studies More junior researchers had to build working relationships; this was particularly challenging for early-career female researchers in male-dominated hierarchies Building these relationships was further challenged by the frequent transfer of senior NTP officials, e.g. six different NTP Line Directors in Bangladesh over the study period Challenging for researchers to stay within their research role and not influence implementation
Step 5: Making strategic choices to support vertical scale-up (institutionalization)	Focusing on health system levers most amenable to change (Ra, Co) Identification of a mix of rural, urban, public, private, large and small learning sites (O, R, Co) Workshops and presentations at key events (C, Co)	Insights of “insider researchers” were valuable in ensuring compatibility and relevance of scale-up strategies In Bangladesh and Nepal, learning sites were agreed upon with NTP at national (Bangladesh) and municipal level (Nepal), but more limited ownership of implementation by NTPs Detailed knowledge of NTPs by the research team was key in identifying the most strategic events to engage and seizing opportunities to engage and influence in e.g. Chest Society conference and inter-provincial and inter-district meetings in Pakistan; NTP Technical Working Group and WHO events in Nepal; coordination workshops/meetings with National Tobacco Control Cell, and Noncommunicable Disease Control Programme, Ministry of Health and Family Welfare in Bangladesh Rapid recognition of the need to engage at municipal level within the new federal context of Nepal to identify learning sites and train, as trainers supported ownership and integration of the intervention at the municipal level
Step 6: Making strategic choices to support horizontal scale-up (increased coverage)	Sharing findings, including costs, from learning sites via policy briefs and workshops (O, Ra, T) Way Forward workshops with NTP, donor and NGO stakeholders in all three countries to build on findings to plan scale-up beyond learning sites (C, O, R) Feeding into TB strategic planning processes (R) Engaging global policy-makers (WHO, United Nations Development Programme, Tobacco Control) and International Union against TB and Lung Disease (C, R)	Inclusion of private sector and NGO providers to enhance testability (T) within different contexts vital within the pluralistic health system Collection of cost data valued by decision-makers within NTPs Multiple channels are needed for dissemination e.g. “Way Forward” workshops, policy briefs and availability of all materials in Urdu, Bengali and Nepali Dissemination most effective when linked to forward planning Integration of tobacco cessation within TB programmes gaining global traction, but TB & Tobacco advisors working in silos. Growing recognition of the need for tobacco indicators within Global Fund proposals and monitoring Engagement of senior researchers in the team in health sector planning (e.g. in Bangladesh: annual programme review (APR) and midterm review (MTR) of the 4th Health Sector Programme of Bangladesh, road map to make Bangladesh tobacco-free by 2040). In Pakistan, engagement in processes to develop the 2020–2023 strategic plan, which highlighted the success in learning sites and emphasized tobacco cessation. In Nepal, engagement in the development of the NTP’s National Strategic Plan, and the funding proposal to GFATM helped to advocate and incorporate research findings to support horizontal scale-up In Nepal, embedding research within a government in transition to federalization was challenging, but building alliances within and beyond NTP provided opportunities for horizontal scale-up
Step 7: Determining the role of diversification	Emphasizing core elements of the interventions within the health worker guide which accompanies the intervention materials (E, Co) Encouraging adaptation to context (Co)	Tension between staying focused on delivery of the existing intervention and requests from learning sites to extend the work, e.g. to include community education campaigns on tobacco, to use within multidrug-resistant (MDR) TB programmes, greater emphasis on smokeless tobacco Adaptation (e.g. group sessions in Pakistan’s busy clinics) and personalization of the delivery of the intervention helped ownership and adoption of the intervention
Step 8: Planning actions to address spontaneous scale-up	Videos and materials freely available online to encourage spontaneous scale-up (E, Co) Link to online materials in all printed intervention materials (E, Co) All materials in multiple languages (Urdu, Bengali, Nepali and English) (E, Co)	Organizations may take the initiative but only implement part of the intervention; e.g., Noncommunicable Disease Control (NCDC) Programme of Bangladesh endorsed the leaflet and printed and distributed nationally (from their own budget) Materials adapted for use in large private TB providers in Pakistan In Nepal, some intervention materials (i.e. posters and leaflets) were considered as NTP resources and so supplied to health facilities through NTP
Step 9: Finalizing scaling-up strategy and identifying next steps	Way Forward workshops with national TB programme policy-makers designed to identify next steps (C, R, Co)	Challenges agreeing on next steps within federal context of Pakistan, and within context of structural reorganizations in newly federal Nepal Importance of close engagement within national NTP strategy processes e.g. greater coordination with NGOs (e.g. Bangladesh Rehabilitation Assistance Committee [BRAC]) and development partners (e.g. Global Fund) in Bangladesh More work at global level needed to shape indicators (e.g. Global Fund)

^a^ExpandNet’s “CORRECT”: C = credible; O = observable; R = relevant; Ra = relative advantage; E = easy to use; Co = compatible; T = testable (WHO 2020 p. 17 [20])

### Strategy 1: simple and adaptable intervention

The facility and qualitative data shed light on the extent and nature of implementation of the intervention with TB patients. Table 6 shows the numbers of smokers identified compared to the expected number of smokers according to standardized WHO estimates and the proportion advised to quit.

**Table 6:** Smokers identified and advised on cessation among all TB patients from routine records

Country: WHO smoking prevalence (2017)	Bangladesh Male (M): 41.4%; female (F): 1.4%; all: 21.3%				Nepal M: 33.7%; F: 8.8%; all: 21.3%	Pakistan, Khyber Pakhtunkhwa (KP) Province M: 31%; F: 2.8%; all: 16.9%				
Learning sites	Narayanganj	Gazipur	Dhaka	Bangladesh total	Kathmandu Lalitpur	Abbottabad	Kohat	Mardan	Peshawar	KP Province total
Number of facilities	5	5	5	15	18	9	10	12	28	59
Data collection period	6 months	3 months			3 months	6 months				
Total patient numbers from routine data over study period	2808 (M: 1551; F: 1257)	1329 (M: 800; F: 529)	1234 (M: 711; F:523)	5371 (M: 3062; F:2309)	288 (M: 148; F:140)	851 (M: 428; F: 423)	580 (M: 283; F: 297)	1081 (M: 554; F: 527)	2577 (M: 1302; F: 1275)	5089 (M: 2567; F: 2522)
Male TB patients, monthly mean	259	267	237	763	49	71	47	92	217	427
Female TB patients, monthly mean	210	176	174	560	47	71	50	88	213	422
Total TB patients, monthly mean	468	443	411	1322	96	142	97	180	430	849
Patients asked about tobacco use, monthly mean	468 (100%)	443 (100%)	411 (100%)	1322 (100%)	96/96 (100%)	142/142 (100%)	97/97 (100%)	180/180 (100%)	430/430 (100%)	849/849 (100%)
Male smokers identified among all TB patients, monthly mean	130/259 (50.1%)	110/267 (41.1%)	75/237 (31.6%)	321/763 (42.1%)	11/49 (22.4%)	21/71 (29.6%)	6/47 (13.1%)	8/92 (8.9%)	27/217 (12.4%)	62/427 (14.5%)
Expected male smokers based on WHO 2017				316/763 (41.4%)	16.5/49 (33.7%)					132.37/427 (31%)
Female smokers identified among all TB patients, monthly mean	0.33/210 (0.2%)	0/176 (0%)	0/174 (0%)	0.33/560 (0.06%)	4/47 (8.5%)	0.5/71 (0.7%)	0/50 (0%)	0/88 (0%)	0/88 (0%)	0.5/422 (0.1%)
Expected female smokers based on WHO 2017				8/560 (14.3%)	4.14/47 (0.088)					11.816/422 (2.8%)
Total smokers identified among all TB patients, monthly mean	130.33/468 (27.8%)	110/443 (24.7%)	75/411 (18.2%)	315.33/1322 (23.9%)	15/96 (15.6%)	21.5/142 (15.1%)	6/97 (6.1%)	8/180 (4.4%)	27/430 (6.3%)	62.5/849 (7.4%)
Expected total smokers based on WHO 2017				324/1322 (24.5%)	20.64/96 (21.5%)					144.186/849 (16.98%)
Ratio of identified versus expected smokers (95% CI)				0.97 (0.86–1.07)	0.73 (0.36–1.10)					0.43 (0.33–0.54)
Patients given cessation advice, monthly mean	130.33/130.33 (100%)	110/110 (100%)	75/75 (100%)	1322/1322 (100%)	15/15 100%	21.5/21.5 (100%)	6/6 (100%)	8/8 (100%)	27/27 (100%)	62.5/62.5 (100%)

In Bangladesh and Nepal, the health workers identified a lower number of smokers than would be expected based on WHO (2017); however, these differences were not statistically significant. In Pakistan, the ratio of identified versus observed shows statistically significantly few smokers identified (ratio 0.43, 95% CI 0.33–0.54) than would be expected given WHO estimates of prevalence. The qualitative findings point to a combination of patient, health worker and site factors. Several health workers mentioned the need to build rapport with their patients before asking about tobacco use, often over a number of visits by the patient to collect their TB medicines. This was despite indicating a high level of confidence in asking about tobacco use in the post-training questions, with all participants scoring at least 4 out of a potential 5, for totally confident, on the questionnaire Likert scale. This did differ depending on the gender of the provider, with male providers feeling more confident to ask male patients, and female health workers to ask female patients.

Once patients had been identified, the routine reporting forms in all countries indicated that all patients had been given support to quit. The qualitative findings noted use of the flipbook and desk guide. However, the data also shed light on how only minimal advice was often given. Many health workers reported challenges in delivering the behaviour support as per the intervention design, with health workers in busy clinics and urban settings rarely able to spend the time required to go through every page of the flipbook:

*I’ve only ever used the flip chart for one patient. My patients cannot spend much time, as all of them are workers. They take a short break from work to come, so I cannot explain to them in detail.* (NP8: Female TB health worker, public facility, Nepal).

Many health workers felt they had been able to internalize the key messages and could deliver these without using the flipbook, filling in any gaps in subsequent patient consultations. In the busiest learning sites in Pakistan, health workers had adapted the intervention by delivering it to groups of patients.

Where our learning sites included both public and private facilities, the qualitative findings identified similar constraints across both settings, although private providers also mentioned the challenges of balancing implementation of the intervention with the specific requirements of their own organizations:

*It can be difficult because we have our own rules here and we cannot go above a certain limit [of interventions delivered].* (PK19: Male TB health worker, private facility, Pakistan)

### Strategy 2: integration of cessation within routine training

The NTBs identified staff at national and subnational levels to be trained as trainers in each country. The training of trainers (ToT) workshops took between 3 and 6 hours. During these ToT sessions, participants identified future events, such as district quarterly and monthly meetings to deliver the 1–2-hour training session with TB health workers. As shown in Table 7, the numbers of trainers trained varied between contexts, depending on the level of engagement of NTPs. The extent to which these trainers then trained TB health workers also varied. In Nepal, while eight NTP and municipal public health officers were trained as trainers, only two provided training to health workers in the subsequent training sessions. In both Bangladesh and Pakistan, trainers expanded their training sessions beyond the initial learning sites to train a further 32 TB health workers in Bangladesh and health workers in all 121 facilities in KP Province by the end of 2020.

**Table 7:** ToT and TB health worker change in capability for cessation support

	NTP staff trained as trainers	TB health workers trained	Capability questionnaire		
			Before training Mean (minimum, maximum)	After training Mean (minimum, maximum)	% Change (95% confidence interval [CI])
Bangladesh	4	37: 5 in learning sites Follow-on training provided by research team and NTP trainers to 32 health workers in addition to training sites	97% (min: 93%; max: 100%)	99% (min: 99%; max: 100%)	3% (95% CI: −0.6 to 6%)
Pakistan	16	115 health workers trained in 4 districts: 55 doctors, 56 DOTS facilitators, 4 district data assistants Follow-on training: all TB staff in 121 facilities trained by 2020	70% (min: 31%; max: 99%)	86% (min: 21%; max: 100%)	16% (95% CI: 13–19%)
Nepal	8	17: 11 TB health workers and 6 public health officers No follow-on training beyond learning sites	59% (min: 36%; max: 86%)	81% (min: 40%; max: 94%)	22% (95% CI: 16–28%)
Combined for all 3 countries	28	169 trained initially 153 received follow-on training	69% (min: 31%; max: 100%)	86% (min: 1% max: 100%	17% (95% CI: 14–20%

Qualitative observations of the training sessions highlighted the value of the videos in helping to maintain consistency in messages delivered and provide relevant demonstrations of rapport-building and clear communication. Participants were observed to be more engaged and motivated in sessions where the trainers were able to use interactive methods and relate material to the realities of routine TB services.

Mixing different levels of seniority was practically necessary in several training sessions; however, this undermined participation:

*It should have been a bit more interactive because I did not see anyone from lower levels of staff participating. It was mostly one-sided. Doctors did participate a bit, but it was not adequate.* (PK10: Male, senior district manager, Pakistan)

The need to keep the training short did present challenges in ensuring interactive delivery, but this was seen by senior managers as vital for scale-up of the intervention:

*It would not be possible to include a half-day session within the current training programme. Rather, a 1-hour interactive session can be introduced.* (BD8: Female, senior national TB manager, Bangladesh)

Despite the short duration of training and the challenges in quality, participants’ questionnaire scores before and after the training showed an increase in their confidence to deliver cessation (see Table 7). Qualitative interviews highlighted areas of new knowledge for many participants, including increased understanding of the health dangers of smokeless tobacco.

Despite increasing their knowledge and confidence in providing cessation support, health workers in all three countries emphasized their preference for longer sessions and regular refresher training. While this was in part due to the high turnover of staff, training was also perceived as a form of incentive.

### Strategy 3: including tobacco use in recording, monitoring and supervision

Following a brief explanation of how to complete the revised recording forms during the training, the TB health workers were asked to use the revised version of the forms with the three new tobacco columns for a period of 6 months (January to June 2019). In Pakistan, the provincial office required health workers to complete the revised forms instead of the existing NTP forms (Forms TB01, 02 and 03). In Bangladesh and Nepal, TB health workers had to complete both the standard TB programme form and the revised form. In Nepal and Bangladesh, this process took some time to negotiate and establish, and ultimately, the revised forms could only be trialled for three months.

The qualitative findings indicate that TB health workers did not find the additional three columns a burden to fill, and some mentioned that it acted as a reminder to raise tobacco use as an issue with their patients:

*There have been changes in the new register. So, when we need to fill up the form for registration, we need to ask about their smoking status. Even if we forget, since it is there in the register, we have to ask the patients.* (NP7: Female TB health worker, public facility, Nepal)

Beyond monitoring tobacco cessation within the TB programme, and reminding health workers to ask about tobacco use, the reporting forms provided the basis for supervision within the facility and from district and provincial level.

*Being an in-charge [facility manager] it is my duty that I go and check if they are actually doing it. I check their data or I observe how they do the counselling of a patient who is a smoker and ask them to do it in front of me and wherever I see a deficiency I should rectify it.* (PK1: Male TB manager, public facility, Pakistan)

In Pakistan, the Provincial TB Programme adapted their supervision checklist to note any training on tobacco cessation and to assess the completeness of recording for tobacco status and cessation advice given. However, the qualitative findings highlighted how supervision visits from the district office to the facility level were often infrequent and focused on checking data in the registers. The influence of supervision was particularly noted, with several facility managers, including those in the private sector, revealing that they only changed to the new reporting forms and began using the intervention materials following a visit by the provincial TB focal person.

During the implementation of this study, the transition to a federal system was underway in Nepal, and the changes in personnel, systems and roles of different government bodies, particularly between ministry and municipal levels, undermined attempts to include tobacco cessation within supervision and monitoring mechanisms:

*All the staff are in confusion on where we will be and what will we do. We are functioning under both the Health Office and the municipality, with instructions coming from both bodies. We are in a dilemma on what we should do because of this...”* (NP4: Male TB manager, public facility, Nepal)

Qualitative interviews and observations highlighted the challenges of influencing supervision practices within the context of the public private partnerships in all three countries. In Bangladesh, where TB services are delivered by multiple providers, including the government, NGOs and private providers, challenges were identified in developing and implementing supervision guidelines that would be used by all.

### Strategy 4: embedding research within the TB programmes

The extent to which we were able to embed the research within TB programmes differed across the countries. A key component of this strategy was the use of “insider researchers”. In Pakistan, a senior member of the national-level CU (coauthor RF) was also a coinvestigator in the TB & Tobacco Consortium, and a senior manager within the KP provincial TB programme (coauthor MD) was involved in data collection and analysis for the study. In Bangladesh and Nepal, the TB & Tobacco Consortium researchers relied upon their existing relationships at national and district levels. The frequent turnover of key staff in both Bangladesh and Nepal further undermined progress towards ownership of the implementation and scale-up process.

The qualitative interviews highlight how in Bangladesh and Nepal, while staff were positive about the intervention, it was clearly seen as an initiative emanating from the research organizations, not an initiative of the NTPs. In contrast, in Pakistan, the provincial TB programme (KP) felt strong ownership, as evidenced by the regular supervision, subsequent rollout of training and revised reporting forms to all 121 TB facilities in the province. Gaining national-level support for the intervention and revision of policies, guidelines and reporting forms was more challenging, particularly in the context of Pakistan’s federal system whereby each provincial TB programme must agree to any changes. Having a senior member of the national-level CU as a coinvestigator of the project was invaluable in influencing other provincial TB programmes. By the end of 2019, while not all TB forms had been revised to include the tobacco columns, the TB01, the form held by the patients and brought to each consultation, had been revised to include tobacco status, and these revised forms will be rolled out nationally.

In Nepal, the timing of our study coincided with organizational changes due to federalization making it challenging to embed the research within the NTP. Despite the continual engagement of the research team, policy support and resources for tobacco cessation were minimal within the TB sector plan [36].

*[To date] the government has not allocated [any financial resources], nor has the international partner, Global Fund, allocated any amount for the TB & Tobacco Programme.* (NP2: Male technical officer, national TB centre, Nepal)

Given the challenges of embedding research when government organizations are undergoing extensive structural change, the team in Nepal used tactics of leveraging the support of others including WHO and sub-recipients of The Global Fund to Fight AIDS, Tuberculosis and Malaria (GFATM, and seizing opportunities to engage with NTP technical working groups. These efforts were realized when the independent team evaluating NTP recommended greater integration of tobacco cessation within the TB programme, resulting in the inclusion of indicators on tobacco use and advice within national reporting forms and training for cessation support added to NTP routine training.

In Bangladesh, the team engaged closely with key government stakeholders in the Tobacco Control Cell and the NCDC department as well as the NTP to stimulate engagement with the project. Engaging with, and facilitating communication between, these national government departments was an important strategy for progressing scale-up and helped overcome challenges of relying on the support of one or two champions, particularly given the frequently changing TB programme officials, including NTP directors. There are some indications that these approaches acted as a catalyst for scale-up, as the NCDC department used their own resources to print the intervention leaflet and disseminate it nationally. However, the pluralism of providers and the multiple donor-funded vertical programmes within Bangladesh’s health sector were a further barrier to progressing scale-up:

*What is needed is coordinated efforts from everyone. The major obstacle is non-coordination of the operational plans and among DPs [development partners]. We have so many vertical programmes without any coordination. Leadership is another problem. Change in staff within different units every 6 months to a year slows down the progress of the work.* (BD8: Female, senior manager, NTP, Bangladesh)

Embedding the research within the three countries required significant flexibility and resourcefulness to tailor tactics to the complex health systems with multiple providers and donors, frequently changing personnel and organizational structures.

### Estimated costs

The estimated costs over the 6-month implementation within the learning sites in each country, including costs per patient, are shown in Table 8.

**Table 8:** Estimated costs (USD) of implementation in the learning sites over 6 months by country

Country	Number of facilities	Total TB patients	Smokers identified	Total personnel cost for training	Total personnel cost for intervention delivery	Total cost for intervention materials	Cost per TB patient
		(6 month estimates)					
Bangladesh	15	7934	1885	$1196	$1047	$1473	$0.5
Nepal	18	576	90	$375	$92	$143	$2.8
Pakistan	59	5089	375	$3041	$687	$3746	$1.5

Per-patient programme costs were US$ 0.5 in Bangladesh, US$ 2.8 in Nepal and US$ 1.5 in Pakistan. While the costs of intervention delivery would increase with the number of patients treated, the training costs include the costs of training the trainers which can be considered as a one-off cost, hence the average costs per patient could potentially be reduced over time. These costs were shared with TB programme decision-makers in policy briefs and as part of a series of workshops to facilitate further planning for scale-up.

## Discussion

**Fig. 1:**
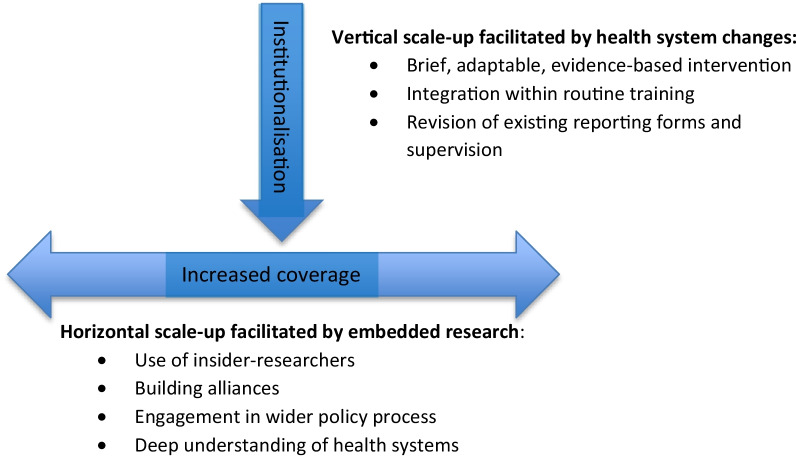
Strategies to support vertical and horizontal scale-up

The four strategies were key in supporting vertical (institutionalization) and, to a lesser extent, horizontal scale-up (increased coverage) of the tobacco cessation intervention (see Fig. 1). In summary, the strategies had the following impact: (1) Reporting forms indicated that all identified tobacco users were advised to quit. TB health workers did not identify all tobacco users; qualitative data showed that health workers used the materials, although not always as intended. Materials were disseminated by NTPs beyond the learning sites in all three countries, indicating some level of horizontal scale-up. (2) Training was effective in building TB health worker confidence and knowledge to deliver cessation support to patients and, as an indication of horizontal scale-up, was delivered beyond the learning sites in Bangladesh and Pakistan. (3) The addition of reporting items on tobacco use and cessation advice to routine reporting forms was feasible for TB health workers to use in everyday practice and informed revisions to national reporting forms in Pakistan. The per-patient programme costs were low in all three countries, with the highest cost of US$ 2.8 in Nepal due primarily to low patient numbers. Costs would reduce with horizontal scale-up, and a stepwise expansion could be considered to spread costs over a few years if needed.

There was much evidence of use of the intervention materials and of adaptation to fit social and cultural norms and to implement within busy TB clinics. Given the challenges of relying on self-report routine data, we cannot draw conclusions on the impact of the reduced and adapted intervention on cessation at 6 months. While the simplification of the intervention is necessary to facilitate both vertical and horizontal scale-up and has been identified as a predictor of successful scale-up [21, 37, 38], the challenge for those developing interventions and planning their implementation is to determine how far the intervention can be reduced and adapted without undermining its effectiveness. It has been argued that there may not necessarily be a “voltage drop” in the effectiveness of interventions from efficacy trial to scale-up [39]. Further, a less efficacious intervention with more reach may have a greater impact then a highly efficacious intervention with less reach. Research assessing the effect of the adaptation of interventions during scale-up is limited [40], and such studies would be a valuable contribution to the evidence base on sustainability. Approaches such as the Dynamic Sustainability Framework emphasize that sustainable scale-up requires continual learning and adaptation to improve the fit of interventions to local settings and contexts, and this enables ongoing improvement as opposed to diminishing outcomes over time [39]. Increasing this level of fit is also a key element in the diffusion of innovations [41].

We found that changes to reporting forms encouraged implementation, provided programme-level data on implementation and acted as a basis for, albeit limited, supervision. The term “checklist effect” [42] has been coined to explain how the process of recording data triggers intervention implementation. Other studies highlight how changes to reporting forms need to be considered early in the intervention design process, or else can undermine attempts at implementation, as found in the assessment of institutionalization of evidence-based newborn health interventions in Bangladesh [22]. In high-income contexts, measurement of outcomes over time has been identified as supporting sustainability of the practice [39, 43].

Influencing progress towards horizontal scale-up of coverage beyond the learning sites was particularly challenging, as has been found for many other public health interventions [20, 21]. Our fourth strategy of embedded research, while valuable for vertical scale-up, proved particularly important as a catalyst to horizontal scale-up or increased coverage. The inclusion of “insider researchers” [26] in Pakistan may well have stimulated this level of commitment. This has been identified as a key strategy for embedded health policy and systems research [26, 27]. The importance of champions and strong leadership is a consistent theme within scale-up models and frameworks [21, 38, 44], as well as being identified in step 2 of the ExpandNet process [20]. However, the experiences from Bangladesh and Nepal highlight that relying on champions alone is not sufficient, particularly where health systems are undergoing major organizational change, as in Nepal, and have frequent senior staff turnover and multiple providers, as in Bangladesh. Deploying multiple strategies, including building alliances with other health system actors and engaging in appropriate fora, helped scale-up in these contexts. Pursuing these strategies in Nepal and Bangladesh was possible due to the close partnership between the research teams, NTPs and the wider health system. These long-standing connections provided the researchers with the “deep knowledge” required to build effective networks and take advantage of opportunities to influence scale-up [45].

Our approach of focusing on four clear strategies accords with Milat’s (2015) review, which identified a well-defined scale-up strategy as key [21]. Finding the most appropriate mix of strategies for the context is fundamental to steps 5 and 6 in the ExpandNet framework. While ExpandNet presents the process of scale-up as a series of linear steps, our experience emphasizes the need for a flexible, iterative approach. While it is helpful to identify clear strategies at the start of the process, flexibility to change course is also needed to respond to the complexity of health systems in dynamic contexts, and resonates with Greenhalgh et al.’s (2019) recommendation to use a combination of an implementation science structured and phased approach with the flexible and adaptive approach advocated by complexity science and an understanding of the social aspects of implementation [46].

*Contribution to the literature:*

- The focus of ExpandNet’s nine-step framework on vertical and horizontal scale-up helps guide planning and analysis of scale-up, although strategies should not be assumed to be linear “steps”.
- Identifying priority system-level changes, such as to routine reporting and training, in partnership with health system actors was a prerequisite to institutionalization of the intervention, that is, vertical scale-up.
- Use of insider researchers, alliance-building, seizing opportunities for engagement in wider policy processes, and building relationships with decision-makers were effective tactics for embedding research.
- When dynamically applied, considering health system complexity, this embedded approach can increase intervention coverage, that is, horizontal scale-up.

*Strengths and limitations* The short duration of the study limited our ability to follow up patients to identify the proportion of smokers who were abstinent at the 6-month end of their TB treatment. Given the challenges of self-reported measures of cessation, biochemical validation of these quits would also have been necessary to assess the effectiveness of the scaled-up intervention within the learning sites [47]. A further limitation was that estimated costs did not include administrative activities, small logistics items, overheads or salary on-costs which may vary across contexts and should be considered when budgeting for scale-up.

Our study has several strengths, including the assessment of specific strategies in three different country contexts which included a range of public, private and NGO providers. The use of mixed methods and the CFIR and ExpandNet [20] framework helped guide our analysis of the steps towards scale-up, drawing on the multiple perspectives of implementation, complexity and social science.

## Conclusions

Establishing a scale-up strategy which included a simplified and adaptable tobacco cessation intervention and prioritized health system changes to reporting forms, training and supervision was key to institutionalizing cessation support for people with TB. Increasing coverage of the intervention was more challenging; this worked well when TB programme staff were also members of the research team and were embedded within the health system. While many scale-up frameworks are presented as a series of steps, we found a flexible and responsive approach with iterations between the steps was needed over a long time period. Following clear strategies to make changes to TB programmes can enable routine delivery of cessation support to TB patients. These strategies are inexpensive and, with concerted efforts from TB programmes and donor partners, tobacco cessation can be institutionalized and scaled up.

## Data Availability

The data used and/or analysed during the current study are available from the corresponding author on reasonable request.

## Abbreviations

CU: Common Unit for TB, HIV and Malaria, Government of Pakistan
CFIR: Consolidated framework for implementation research
DP: Development partners
GFATM: The Global Fund to Fight AIDS, Tuberculosis and Malaria
KP Province: Khyber Pakhtunkhwa Province, Pakistan
NCDC: Noncommunicable Disease Control department, Government of Bangladesh
NGO: Nongovernmental Organization
NTP: National TB programme
ToT: Training of trainers
TB: Tuberculosis
